# Supplementary material for: Flexible and cost-effective genomic surveillance of P. falciparum malaria with targeted nanopore sequencing
Source: Nat Commun. 2024 Feb 15;15:1413. doi: 10.1038/s41467-024-45688-z (PMC10869361; doi:10.1038/s41467-024-45688-z)
Supplement: Supplementary file 3 — Reporting Summary [file 41467_2024_45688_MOESM3_ESM.pdf]

nature portfolio

Corresponding author(s): Jason A. Hendry

Last updated by author(s): Jan 22, 2024

Reporting Summary

Nature Portfolio wishes to improve the reproducibility of the work that we publish. This form provides structure for consistency and transparency in reporting. For further information on Nature Portfolio policies, see our Editorial Policies and the Editorial Policy Checklist.

Statistics

For all statistical analyses, confirm that the following items are present in the figure legend, table legend, main text, or Methods section.

n/a	Confirmed	
☐	☒	The exact sample size (*n*) for each experimental group/condition, given as a discrete number and unit of measurement
☐	☒	A statement on whether measurements were taken from distinct samples or whether the same sample was measured repeatedly
☐	☒	The statistical test(s) used AND whether they are one- or two-sided<br>*Only common tests should be described solely by name; describe more complex techniques in the Methods section.*
☐	☒	A description of all covariates tested
☒	☐	A description of any assumptions or corrections, such as tests of normality and adjustment for multiple comparisons
☐	☒	A full description of the statistical parameters including central tendency (e.g. means) or other basic estimates (e.g. regression coefficient) AND variation (e.g. standard deviation) or associated estimates of uncertainty (e.g. confidence intervals)
☐	☒	For null hypothesis testing, the test statistic (e.g. *F*, *t*, *r*) with confidence intervals, effect sizes, degrees of freedom and *P* value noted<br>*Give P values as exact values whenever suitable.*
☐	☒	For Bayesian analysis, information on the choice of priors and Markov chain Monte Carlo settings
☒	☐	For hierarchical and complex designs, identification of the appropriate level for tests and full reporting of outcomes
☐	☒	Estimates of effect sizes (e.g. Cohen's *d*, Pearson's *r*), indicating how they were calculated

Our web collection on statistics for biologists contains articles on many of the points above.

Software and code

Policy information about availability of computer code

Data collection	No software was used as part of data collection.
Data analysis	Basecalling used Guppy v5.0.11 (R9.4.1.) or Dorado v0.34 (R10.4.1). Mapping used minimap2 v2.24-r1122. Downstream analysis made use of samtools v1.17 and Clair3 v1.0.4. Pipelines and figures were generated in python and are available here: https://github.com/JasonAHendry/nomadic2

For manuscripts utilizing custom algorithms or software that are central to the research but not yet described in published literature, software must be made available to editors and reviewers. We strongly encourage code deposition in a community repository (e.g. GitHub). See the Nature Portfolio guidelines for submitting code & software for further information.

Data

Policy information about availability of data

All manuscripts must include a data availability statement. This statement should provide the following information, where applicable:

- Accession codes, unique identifiers, or web links for publicly available datasets
- A description of any restrictions on data availability
- For clinical datasets or third party data, please ensure that the statement adheres to our policy

Reads were mapped to release 52 of the P. falciparum reference genome for strains 3D7 (https://plasmodb.org/common/downloads/release-52/Pfalciparum3D7), Dd2 (https://plasmodb.org/common/downloads/release-52/PfalciparumDd2), GB4 (https://plasmodb.org/common/downloads/release-52/PfalciparumGB4) and

HB3 (https://plasmodb.org/common/downloads/release-52/PfalciparumHB3) downloaded from PlasmoDB; and to the GRCh38 human reference genome (https://www.ncbi.nlm.nih.gov/assembly/GCF_000001405.26/) downloaded from NCBI. Sequence data is available for download from NCBI's Sequence Read Archive (https://www.ncbi.nlm.nih.gov/sra) under the accession PRJNA956048.

Research involving human participants, their data, or biological material

Policy information about studies with human participants or human data. See also policy information about sex, gender (identity/presentation), and sexual orientation and race, ethnicity and racism.

Reporting on sex and gender	Patient sex, but not gender was recorded in the study. Analysis in this manuscript was not not disaggregated as it was not relevent to the genetic profile of the parasite.
Reporting on race, ethnicity, or other socially relevant groupings	There were no race, ethnicity, or other socially relevant groupings.
Population characteristics	There were no covariate-relevant population characteristics.
Recruitment	All individuals presenting with uncomplicated P. falciparum malaria to a health facility in the study were eligible for enrollment, with two exceptions due to drug contraindications. Firstly, children under 6 months of age (>5 kg weight) and women aged 12-18 who could be pregenant and to whom a pregnancy test could not be asked for due to local culture / customs.
Ethics oversight	Ethical permission was obtained from the ERES Converge IRB (Lusaka, Zambia), the Zambian Ministry Of Health and the National Health Research Authority to conduct the study.

Note that full information on the approval of the study protocol must also be provided in the manuscript.

Field-specific reporting

Please select the one below that is the best fit for your research. If you are not sure, read the appropriate sections before making your selection.

☒ Life sciences ☐ Behavioural & social sciences ☐ Ecological, evolutionary & environmental sciences

For a reference copy of the document with all sections, see nature.com/documents/nr-reporting-summary-flat.pdf

Life sciences study design

All studies must disclose on these points even when the disclosure is negative.

Sample size	For experiments using mock samples, sample size was the outcome of making sure all relevant conditions were included with multiple example strains. For field samples, sample sizes were dictated by access on the field team. No power analyses or statistical methods were used to determine sample size, as we were looking to validate performance of our assay rather than measure a biological or epidemiological effect with the samples.
Data exclusions	No exclusions.
Replication	For coverage analyses, each mock sample was sequenced once with the NOMADS8 and once with NOMADS16 panels. For SNP calling analysis, we included multiple laboratory strains carrying known variants. We created ten in silico replicates for each read depth and laboratory strain when estimating F1 scores. For hrp2/3 deletion analyses, we included triplicates for each laboratory strain (3D7, Dd2 and HB3) and parasitemia-level. Field samples sequenced in Oxford were sequenced twice, once with NOMADS8 and once with the NOMADS16 panels. Field samples sequenced in Zambia were sequenced once, with the NOMADS8 panel.
Randomization	We included multiple conditions in a single experiment (for example, presence and absence of a given variant or deletion) so that covariates were controlled and we can assess the variable of interest.
Blinding	Sequencing experiments were done in batches based on sample collection schedule and the experimenter was aware of which sample set they were working with. Blinding not relevant for our design.

Reporting for specific materials, systems and methods

We require information from authors about some types of materials, experimental systems and methods used in many studies. Here, indicate whether each material, system or method listed is relevant to your study. If you are not sure if a list item applies to your research, read the appropriate section before selecting a response.

Materials & experimental systems

n/a	Involved in the study
☒	☐ Antibodies
☒	☐ Eukaryotic cell lines
☒	☐ Palaeontology and archaeology
☒	☐ Animals and other organisms
☒	☐ Clinical data
☒	☐ Dual use research of concern
☒	☐ Plants

Methods

n/a	Involved in the study
☒	☐ ChIP-seq
☒	☐ Flow cytometry
☒	☐ MRI-based neuroimaging

Plants

Seed stocks	*Report on the source of all seed stocks or other plant material used. If applicable, state the seed stock centre and catalogue number. If plant specimens were collected from the field, describe the collection location, date and sampling procedures.*
Novel plant genotypes	*Describe the methods by which all novel plant genotypes were produced. This includes those generated by transgenic approaches, gene editing, chemical/radiation-based mutagenesis and hybridization. For transgenic lines, describe the transformation method, the number of independent lines analyzed and the generation upon which experiments were performed. For gene-edited lines, describe the editor used, the endogenous sequence targeted for editing, the targeting guide RNA sequence (if applicable) and how the editor was applied.*
Authentication	*Describe any authentication procedures for each seed stock used or novel genotype generated. Describe any experiments used to assess the effect of a mutation and, where applicable, how potential secondary effects (e.g. second site T-DNA insertions, mosiacism, off-target gene editing) were examined.*
